# Bite Force, Occlusal Contact and Pain in Orthodontic Patients during Fixed-Appliance Treatment

**DOI:** 10.3390/dj10020014

**Published:** 2022-01-19

**Authors:** Nicoline Mie Therkildsen, Liselotte Sonnesen

**Affiliations:** Section for Orthodontics, Department of Odontology, Faculty of Health and Medical Sciences, University of Copenhagen, DK-2200 Copenhagen, Denmark; nicolinetherkildsen@gmail.com

**Keywords:** bite force, occlusal contact, pain, orthodontic treatment, minor malocclusion

## Abstract

Previously, bite force, occlusal contact and pain were investigated in orthodontic patients with moderate-to-severe malocclusion, but not in patients with minor malocclusion. The purpose of this study was to investigate changes in bite force, teeth in occlusal contact and pain in orthodontic patients with minor crowding before orthodontic treatment (T0), after bonding (T1), during treatment (T2), post-treatment (T3) and during retention (T4). In total, 27 patients (21 females, 6 males, median age 15.3 years) with neutral occlusion and normal craniofacial morphology were treated with non-extractions and fixed appliances. Differences in the registered data were analysed by a mixed linear model with repeated measures. Bite force and teeth in occlusal contact significantly decreased between T0 and T1 (*p* < 0.0001, respectively) and between T0 and T2 (*p* < 0.01, respectively). Bite force and teeth in occlusal contact significantly increased between T1 and T4 (*p* < 0.05, *p* < 0.0001, *p* < 0.001, respectively) and between T2 and T4 (*p* < 0.05, *p* < 0.0001, *p* < 0.01, respectively). No significant difference in pain was found. The results indicate that bite force and teeth in occlusal contact significantly decreased during treatment and reached baseline level at retention. The findings may prove valuable for informing orthodontic patients with minor malocclusion.

## 1. Introduction

Orthodontic treatment with fixed appliances often starts with a levelling phase using flexible arch wires, in which the maxillary and mandibular dental arches are both levelled. This forms two dental arches with correct position of the teeth in each arch where the dental arches do not necessarily fit together [1,2] and the number of teeth in occlusal contact decreases accordingly. Since bite force is associated with the number of teeth in occlusal contact, the lowest bite force is assumed to coincide with the treatment phase with the fewest number of teeth in occlusal contact [3,4,5]. Accordingly, bite force is assumed to increase after treatment due to the establishment of an increasing number of teeth in occlusal contact and close intercuspidation.

Previous studies have found a change in bite force due to orthodontic treatment, before and after treatment [4,6,7]. In skeletal class I patients with increased horizontal maxillary overjet and crowding, bite force was at its lowest point one week after bonding of fixed appliance. The bite force then increased and reached pre-treatment levels after 6 months of treatment [6]. In addition, it was found that for patients with class I and class II malocclusions, bite force increased immediately after debonding the fixed appliances and increased further after 3 months of retention [8]. This was in disagreement with another study, in which it was found that bite force decreased immediately after debonding in skeletal class II patients [9]. It has also been found that the bite force of patients with posterior cross bite decreased immediately after orthodontic treatment, but increased to the same level as subjects with neutral occlusion after the retention period [7].

Orthodontic treatment also changes the number of teeth in occlusal contact. It was been found that the number of teeth in occlusal contact decreased in skeletal class I, class II and class III orthodontic patients after debonding the fixed appliance and increased again during a retention period [4,10,11,12]. In spite of a long retention period after orthodontic treatment of skeletal class I and class II div. 1 patients, the number of teeth in occlusal contact in adults may not reach the same level as that of an untreated control group [12].

Pain during orthodontic treatment is also reported. Up to 95% of all orthodontic patients may experience pain in association with fixed-appliance orthodontic treatment [13,14]. During orthodontic treatment, pain may occur in association with traumatic impact on the surrounding mucosa and the application of force on teeth [13,15,16,17]. It was found that if pain is present, the intensity of the pain generally peaks 24 to 48 h after the fixed orthodontic appliance is inserted [16,18]. Other studies have found that pain is at its maximum 1–2 weeks after the fixed appliance is inserted [6,19].

As changes in bite force, teeth in occlusal contact and pain are associated with fixed orthodontic appliance treatment and may not normalise after treatment, it is essential to know how these parameters change during treatment of heathy subjects with neutral occlusion who want treatment of minor crowding for aesthetic reasons. This has not been reported in previous studies.

The purpose of the present study was to investigate changes in bite force, teeth in occlusal contact and pain in adolescent and adult patients with neutral occlusion and minor crowding in the anterior region before, during and after fixed-appliance treatment.

## 2. Materials and Methods

### 2.1. Subjects

The study included all patients who began orthodontic treatment at the Orthodontic Section, Faculty of Health and Medical Sciences, University of Copenhagen, Denmark, in the period of November 2014–March 2015, and who met the below inclusion criteria.

#### 2.1.1. Inclusion Criteria

- Healthy adolescents and adults
- Neutral molar occlusion and incisal relationship (overjet between 1 and 5 mm and overbite between 1 and 4 mm)
- Normal craniofacial morphology [20]
- Minor crowding in the anterior region that did not meet the national Danish criteria for malocclusion entailing health risks [21] (crowding of less than or equal to 5 mm in the anterior region)
- Non-extraction treatment with fixed appliance using the straight-wire technique [22]

#### 2.1.2. Exclusion Criteria

- Severe malocclusion traits that met the national Danish criteria for malocclusion entailing health risks [21]
- Craniofacial anomalies and systemic diseases

Orthodontic treatment with fixed appliance in both jaws used American orthodontic brackets or titanium orthodontic brackets, both slot 0.018. The first arch wires in the levelling phase were NiTi wires. After the levelling phase, TMA arch wires or stainless steel arch wires were inserted. Average treatment time was 15.8 months (Table 1).

**Figure 1:**
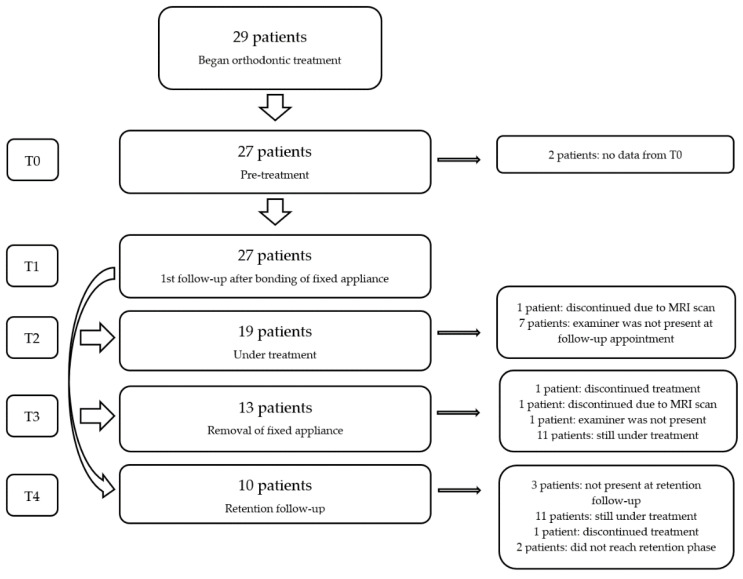
Flowchart.

The sample included 29 patients, but two patients were excluded from the study as they started the orthodontic treatment before the data collection began, and, accordingly, they did not present any pre-treatment data. Thus, the final sample included 27 patients between 12 and 31 years (21 females and 6 males, average age 17.3 years and median age 15.3 years, Figure 1).

This study has been approved by the Danish Data Protection Agency (7. no. 2014-54-0832).

### 2.2. Study Parameters

The following parameters were registered for all patients:

- Maximum unilateral bite force on both sides [23]
- Number of teeth and number of teeth in occlusal contact [3]
- Quantitative pain [24,25]

Study parameters were registered at the following intervals (Table 1):

- Pre-treatment (T0)
- First follow-up after bonding of fixed orthodontic appliance in both jaws (average 3.6 months, T1)
- Duration of treatment (average 13.9 months, T2)
- Fixed-appliance treatment end (average 15.8 months, T3)
- First follow-up after fixed appliance treatment end (average 17.2 months, T4)

The registrations were carried out by the first author, who was trained, and calibrated by the second author, who is an experienced examiner, with an acceptable method error (e.g., [26,27]).

#### 2.2.1. Bite Force Measurement

**Figure 2:**
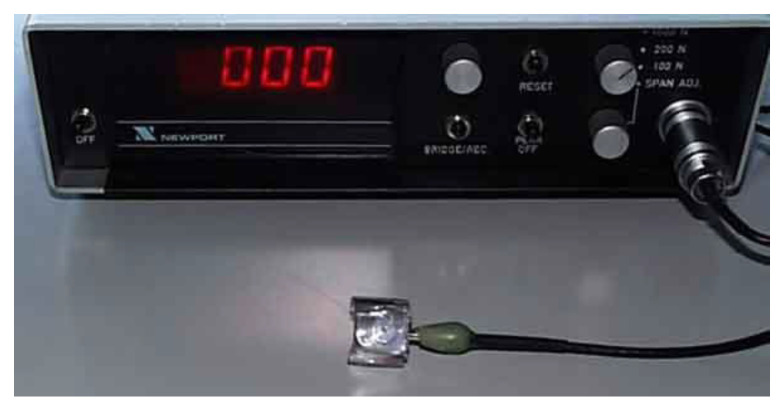
A photograph of the miniature pressure transducer used for the measurement of the bite force.

The maximum unilateral bite force was measured at the first mandibular molars on each side using a miniature pressure transducer [28] (Figure 2). The bite force was measured unilaterally two times on the right side and two times on the left side as stored peak values during maximal effort with the transducer placed on the first mandibular molars. The peak value was registered during 1–2 s of maximum clenching. The bite force was then calculated as the average of the four measurements [23] (Figure 2).

#### 2.2.2. Number of Teeth and Teeth in Occlusal Contact

The number of present teeth and teeth in occlusal contact was registered using standard methods [3]. The number of teeth in occlusal contact was assessed by the patient’s ability to hold a plastic strip between the teeth against a strong pull when the patient’s teeth were firmly clenched [3]. The plastic strip was 0.05 mm thick and 6 mm wide (Hawe Transparent Strips No. 690, straight, Kerr Hawe SA, Bioggio, Switzerland).

Teeth in occlusal contact and present teeth were used to calculate the occlusal support zones according to the Eichner Index [29]. The index divides molar and premolar contact into: A in subdivisions A1–A3 (A1: contact in all four posterior support zones on all the teeth; A2: contact in all four posterior support zones except on one tooth in a support zone; A3: contact in all four posterior support zones except on two or more teeth in the support zones), B in subdivisions B1–B4 (B1: contact in three posterior support zones; B2: contact in two posterior support zones; B3: contact in one posterior support zone; B4: no contact in any posterior support zone, only contact in the anterior region) and C: No occlusal contact. None of the patients was grouped in B4 or C. Therefore, B4 and C are not displayed in the table or in the figure.

#### 2.2.3. Pain Registration

The patients’ subjective pain intensity in the facial region was registered using the Visual Analogue Scale (VAS) [24,25]. VAS is a 10 cm long line with a scale going from 0 to 10, where 0 represents no pain in the facial region and 10 is the worst pain imaginable to the patient [24,25]. The patients were asked to mark their pain intensity on the scale from 0 to 10. The mark was measured to the closest ½ mm (Table 1).

### 2.3. Statistical Analysis

IBM SPSS Statistics 23 Data Editor (Chicago, IL, USA) and SAS (v9.4, SAS Institute, Cary, NC, USA) were used for the statistical analysis. *p* < 0.05 was considered a significant result. Normal distribution was assessed using QQ plots and all continuous variables were normally distributed. The Spearman rank correlation test was used to test association with age. The Wilcoxon test was used to test association with sex. All treatment phases were tested against each other using a mixed-model post hoc test (Bonferroni) in order to see whether there was a significant difference between the treatment phases, i.e., the study parameters between the phases T0-T1, T0-T2, T0-T3, T0-T4, T2-T1, T3-T1, T4-T1, T3-T2, T4-T2 and T4-T3 were tested. A paired Wilcoxon test was used to assess the ranked data on the Eichner Index.

## 3. Results

### 3.1. Descriptive Data at Treatment Start (T0)

The mean values for bite force, number of present teeth and teeth in occlusal contact are shown in Table 1. No significant difference between age and sex was found, except for teeth in occlusal contact and the Eichner Index. Males presented significantly more teeth in occlusal contact and occlusal contact in accordance with the Eichner Index than females (*p* = 0.005, *p* = 0.0068, respectively).

### 3.2. Changes over Time

#### 3.2.1. Bite Force

**Figure 3:**
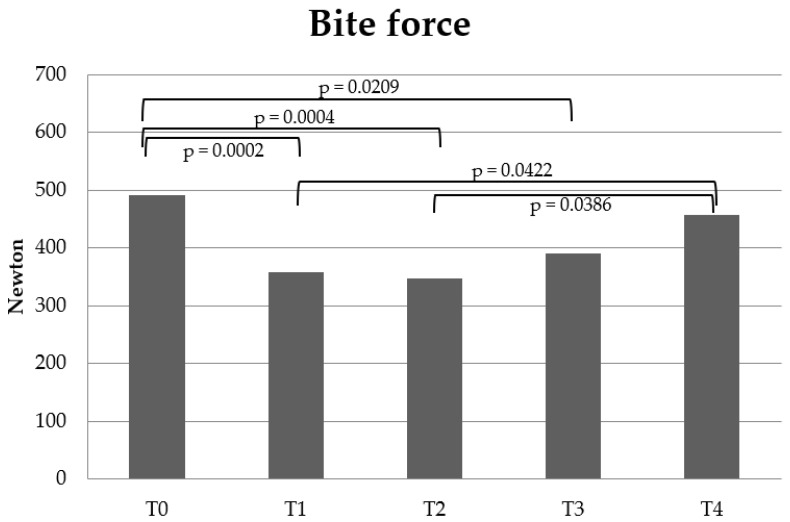
Bite force changes. The chart shows the mean bite force at the different treatment phases: Pre-treatment (T0), 1st follow-up after bonding fixed appliance (T1), during treatment (T2), post-treatment (T3) and at 1st retention follow-up (T4). The significant *p*-values are shown tested by a mixed-model post hoc test (Bonferroni).

Bite force decreased significantly between T0-T1 (*p* = 0.0002), T0-T2 (*p* = 0.0004), and T0-T3 (*p* = 0.0209). Bite force increased significantly between T1-T4 (*p* = 0.0422) and T2-T4 (*p* = 0.0396). There was no significant difference in bite force between T0-T4, T1-T2, T1-T3, T2-T3 or T3-T4 (Figure 3).

#### 3.2.2. Teeth in Occlusal Contact

**Figure 4:**
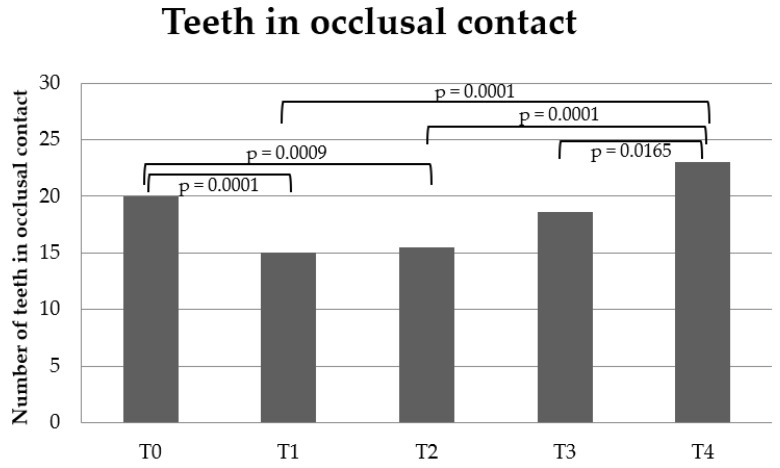
Changes in number of teeth in occlusal contact. The chart shows the number of teeth in occlusal contact at the different treatment phases: Pre-treatment (T0), 1st follow-up after bonding of fixed appliance (T1), during treatment (T2), post-treatment (T3) and at 1st retention follow-up (T4). The significant *p*-values are shown tested by a mixed-model post hoc test (Bonferroni).

**Figure 5:**
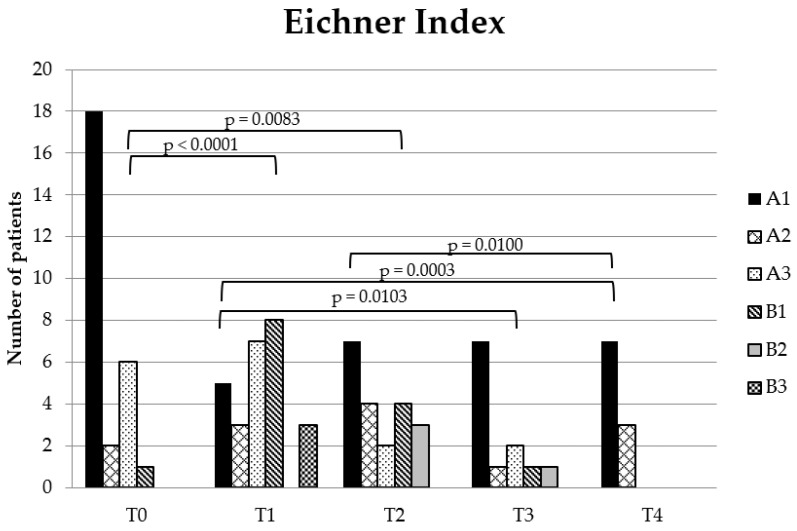
Eichner Index changes. The chart shows the number of patients in the Eichner Index groupings at the different treatment phases: pre-treatment (T0), 1st follow-up after bonding of fixed appliance (T1), during treatment (T2), post-treatment (T3) and at 1st retention follow-up (T4). The significant *p*-values are shown tested by a paired Wilcoxon test.

Teeth in occlusal contact and the Eichner Index decreased between T0-T1 (*p* = 0.0001, *p* < 0.0001, respectively) and T0-T2 (*p* = 0.0009; *p* = 0.0083, respectively). Teeth in occlusal contact increased significantly between T1-T4 (*p* < 0.0001), T2-T4 (*p* < 0.0001) and T3-T4 (*p* = 0.0165), and the Eichner Index increased significantly between T1-T3 (*p* = 0.0103), T1-T4 (*p* = 0.0003) and T2-T4 (*p* = 0.0100) (Figure 4 and Figure 5). There was no significant difference between the other time points.

#### 3.2.3. Pain Intensity

No significant changes in pain intensity between the different treatment phases was found A tendency towards a significant difference between T1-T2 (*p* = 0.0567) could be seen, i.e., an increased pain tendency from T1-T2.

## 4. Discussion

The aim of the present study was to evaluate changes in bite force, teeth in occlusal contact and pain in adolescent and adult patients with neutral occlusion and minor crowding in the anterior region before, during and after fixed-appliance treatment. These parameters have not been reported in previous research in healthy subjects with neutral occlusion who want treatment of minor crowding for aesthetic reasons. In the present study prior to orthodontic treatment, the mean values for the craniofacial morphology [20], bite force and number of teeth in occlusal contact were comparable to previous studies of healthy subjects [3,23,30,31]. Thus, the craniofacial morphology, bite force and teeth in occlusal contract were considered to represent a healthy population prior to orthodontic treatment; accordingly, the patient group in the present study comprised its own control group. In about half of the patients, it was not possible to register the parameters at the last time point in the retention period (T4), which may be a limitation of the present study. As the dropouts were random, mainly due to administrative reasons, it may not be considered a bias. The methods used in the present study were standard validated methods [3,23,25,27,29,32,33,34].

### 4.1. Changes over Time

#### 4.1.1. Bite Force and Occlusal Contact

In the present study, bite force changed during orthodontic treatment. In comparison with pre-treatment (T0), bite force decreased at the first follow-up after the bonding of fixed appliances in both jaws (T1), during treatment (T2) and at the end of orthodontic treatment (T3). There was no significant difference in bite force between pre-treatment (T0) and the first retention follow-up (T4). However, at the first retention follow-up (T4) there was a significantly increased bite force in comparison with the first follow-up after the bonding of fixed appliances in both jaws (T1) and during treatment (T2). This means that the bite force decreased during fixed orthodontic treatment and recovered to pre-treatment levels at the first follow-up after treatment. This is in agreement with previous studies on patients with severe malocclusion traits [4,8], in which it was found that bite force decreased in the pre-treatment-to-post-treatment period and subsequently recovered in the retention phase. Another study on skeletal type I patients with a slightly increased horizontal maxillary overjet and minor crowding, which approximated the present study’s treatment group, found that bite force was at its lowest point one week after the bonding of the fixed appliance, but then slowly increased and was at pre-treatment levels 6 months after treatment began [6]. This is partly in agreement with the findings in the present study, which also showed that bite force decreased after the bonding of the fixed appliance. However, the present study did not find that bite force increased significantly during orthodontic treatment.

It has previously been shown that one of the most important factors associated with bite force is the number of teeth in occlusal contact [3,5]. The present study showed that the number of teeth in occlusal contact was reduced from pre-treatment to the first follow-up after the bonding of fixed appliances in both jaws (T1) and from pre-treatment to during treatment (T2). It increased from both the first follow-up after the bonding of fixed appliances in both jaws (T1) and during treatment (T2) to the first retention follow-up (T4). The occlusal contact assessed by the Eichner Index showed the same pattern. This is in agreement with other studies, which found that the number of teeth in occlusal contact decreased during the orthodontic treatment of patients with malocclusions and increased during the retention period [4,11,12].

In the present study, the variation in bite force reflects the variation in occlusal contact due to the association between bite force and occlusal contact [3,5]. The decrease in occlusal contact during orthodontic treatment and the increase in occlusal contact in the retention period may be explained by the change in intercuspidation during orthodontic treatment. During orthodontic treatment, the teeth are moved and, accordingly, intercuspidation is disrupted. After orthodontic treatment, the teeth settle vertically and the number of occlusal contacts increases during the retention period [11,35].

#### 4.1.2. Pain

In the present study, there was no significant difference in pain intensity during the orthodontic treatment of patients with neutral occlusion and minor crowding in the anterior region. There was a tendency towards increased pain between the first follow-up after the bonding of fixed appliances in both jaws (T1) and during treatment (T2), but in general, the pain intensity was low during the entire treatment.

Previously, it was found for skeletal class I patients with increased horizontal overjet that the prevalence of pain and pain intensity were at their highest 1 to 2 weeks after bonding of a fixed appliance. There was then a general decrease in the prevalence of pain and pain intensity up to the 6 month follow-up during orthodontic treatment [6]. Another study of skeletal class II patients found that the pain intensity increased up to one week after the start of fixed-appliance treatment, but at a one-month follow-up there was no significant difference in pain compared to pre-treatment [19]. It was also found that pain intensity was at its highest 24 to 48 h after the bonding of a fixed appliance [16,18].

Many factors influence pain intensity. In general, pain increases with age [36,37,38] and women experience increased pain intensity compared to men [25,39,40]. Pain intensity may also be associated with different cultures and with ethnicity [41,42]. With regards to orthodontic patients, no significant association between pain intensity and severity of crowding has been found [15,43], but there was a significant association between a patient’s motivation for orthodontic treatment and pain intensity [44]. An explanation for why pain intensity was not affected by orthodontic treatment in the present study may be that the patients were highly motivated to undergo orthodontic treatment.

### 4.2. Clinical Implementation of the Findings

The present study showed that bite force and occlusal contact changed at the first follow-up after the bonding of a fixed appliance and during treatment in comparison with pre-treatment. After treatment, when the fixed appliance was de-bonded and at the subsequent retention follow-up, bite force and occlusal contact were at the same level as pre-treatment. The performance of diagnostics before orthodontic treatment are important in order to provide patients with the best treatment and guidance [45]. When carrying out orthodontic treatment for healthy subjects with neutral occlusal and normal craniofacial morphology with minor crowding, it is important that we do not harm any normal pre-treatment conditions. Accordingly, the results of the present study are important for informing patients before orthodontic treatment.

The results of the present study may also contribute to anchorage considerations. Bite force can inhibit the unwanted vertical extruding forces applied on the teeth during the levelling phase of orthodontic treatment and is considered the patient’s own anchorage [23]. As the present study showed that bite force decreased during treatment, the requirement for anchorage may increase even in patients with neutral occlusion and normal craniofacial morphology. Furthermore, it is often the patient’s own bite force that is required to maintain the obtained occlusion after treatment in the long term, sometimes together with fixed or removable retention appliances [45,46]. Since bite force and occlusal contact in the present study were at the same level after treatment as before treatment, these parameters do not demand increased retention requirements.

## 5. Conclusions

The present study showed that bite force and teeth in occlusal contact significantly decreased during treatment and reached baseline levels at retention. There was no significant difference in pain during or after orthodontic treatment. These normal conditions prior to orthodontic treatment did not change after orthodontic treatment for subjects with neutral occlusion and normal craniofacial morphology. These findings may prove valuable for informing orthodontic patients before treatment of minor crowding for aesthetic reasons, and for treatment considerations in healthy subjects with neutral occlusal and normal craniofacial morphology.

## Figures and Tables

**Table 1:** Bite force, occlusal contact and pain intensity at the different treatment phases.

Variable	T0		T1		T2		T3		T4	
*n*	27		27		19		13		10	
	Mean	SD	Mean	SD	Mean	SD	Mean	SD	Mean	SD
Time from T0 (month)	-	-	3.6	2.5	13.9	6.7	15.8	2.8	17.2	3.3
Bite force	490.8	166.9	358.4	108.1	346.5	107.1	389.9	116.4	457.2	88.3
Teeth in occlusal contact	20.0	4.3	15.0	4.9	15.5	5.4	18.6	4.8	23.0	4.4
Pain intensity (mm)	6.4	13.2	2.6	5.4	10.6	21.3	4.7	9.7	4.7	13.0
Eichner Index										
A1	18	66.67	5	19.2	6	31.6	8	61.5	7	70
A2	2	7.41	3	11.5	4	21.1	1	7.7	3	30
A3	6	22.22	7	26.9	2	10.5	2	15.4	0	0
B1	1	3.7	8	30.8	4	21.1	1	7.7	0	0
B2	0	0	0	0	3	15.8	1	7.7	0	0
B3	0	0	3	11.5	0	0	0	0	0	0

T0: Pre-treatment; T1: First follow-up after bonding of fixed orthodontic appliance in both jaws; T2: During treatment; T3: Day of fixed orthodontic appliance treatment debonding; T4: First follow-up after fixed orthodontic appliance treatment; SD: Standard deviation.

## Data Availability

The data presented in this study are available on request from the corresponding author. The data are not publicly available due to privacy.
